# Influence of internal limiting membrane peeling during idiopathic epiretinal membrane removal: a randomized controlled trial

**DOI:** 10.1038/s41598-025-01987-z

**Published:** 2025-05-20

**Authors:** Bo Hee Kim, Chang Ki Yoon, Kunho Bae, Eun Kyoung Lee, Chan Ho Lee, Dong Ik Kim, Ki Woong Bae, Un Chul Park

**Affiliations:** 1 Department of Ophthalmology, Seoul National University College of Medicine, Seoul, Korea; 2 Department of Ophthalmology, Kangbuk Samsung Hospital, Sungkyunkwan University School of Medicine, Seoul, Korea; 3 Department of Ophthalmology, Hangil Eye Hospital, Incheon, Korea; 4 Department of Ophthalmology, Nowon Eulji Medical Center, Seoul, Korea; 5 Department of Ophthalmology, Seoul National University Hospital, Seoul National University College of Medicine, 101, Daehak-ro, Jongno-gu, Seoul, 03080 Republic of Korea

**Keywords:** Internal limiting membrane, Epiretinal membrane, Recurrence, Metamorphopsia, Randomized controlled trials, Retinal diseases, Outcomes research

## Abstract

In epiretinal membrane (ERM) surgery, the influence of internal limiting membrane (ILM) integrity after ERM removal is underexplored. This study investigated outcomes of idiopathic ERM surgery based on ILM condition following ERM removal and intentional peeling of residual ILM. In this prospective, randomized clinical trial, 102 patients underwent vitrectomy for idiopathic ERM. After ERM removal, ILM status was evaluated using indocyanine green staining. Patients with mostly removed ILM were allocated to group 1 (involuntary peeling), whereas those with partially removed or intact ILM were randomized 1:1 to active peeling (group 2) or non-peeling of residual ILM (group 3). Recurrence of ERM during 12-month follow-up occurred only in group 3 (36.1%), particularly when residual ILM involved the fovea. Best-corrected visual acuity, metamorphopsia, and aniseikonia did not differ among groups; however, group 3 showed significantly lower central macular thickness at all follow-ups (*p* < 0.05) and better macular contour restoration according to retinal thickness profiles. In patients whose residual ILM involved the fovea, non-peeling resulted in less severe vertical metamorphopsia when ERM did not recur. These findings suggest that residual ILM peeling decisions during ERM surgery should consider both anatomical and functional outcomes, as non-peeling favoring anatomical macular recovery but increasing recurrence risk.

**Supplementary Information:**

The online version contains supplementary material available at 10.1038/s41598-025-01987-z.

## Introduction

Idiopathic epiretinal membrane (ERM) is a common macular disease that affects 2% of individuals younger than 60 years and 12% of those older than 70 years^1^. It is characterized by fibrocellular proliferation on the inner surface of the retina that can distort the normal structure of the macula, resulting in decreased central visual acuity, metamorphopsia, macropsia, and micropsia^2,3^.

Pars plana vitrectomy (PPV) and ERM removal using microforceps are considered the standard treatment for symptomatic ERM. However, ERM recurrence is observed in approximately 10–21% of patients after successful surgery, requiring reoperation in 3–6% of patients^4,5^. Therefore, additional peeling of the internal limiting membrane (ILM) has been widely used during ERM surgery to prevent ERM recurrence, because the ILM may serve as a scaffold for cellular proliferation and its peeling ensures complete removal of ERM fragments^6,7^. Several studies have also demonstrated a lower ERM recurrence rate when the ILM was peeled additionally compared to ERM removal alone, while visual outcome did not differ between eyes with and without ILM peeling^8–10^.

However, intentional peeling of the ILM for ERM treatment still remains controversial, because the ILM is the basal lamina connected to the end feet of Müller cells and its peeling may cause functional and mechanical damage to the retina^11,12^. In addition, intraoperative use of dyes such as indocyanine green (ICG) to enhance visualization of the ILM, which is reportedly toxic to the retinal pigment epithelium, may also influence retinal function^13,14^.

During ERM removal, the ILM is peeled off simultaneously with the ERM en bloc or partially^15,16^. However, the influence of ILM integrity after ERM removal has not been considered in most studies that have compared the results of peeling and non-peeling of ILM. Therefore, we aimed to evaluate the results of idiopathic ERM removal surgery according to the ILM condition after ERM removal and intentional peeling of the residual ILM.

## Methods

### Study design and participants

This prospective, randomized, controlled, single-blind clinical trial was conducted at the Seoul National University Hospital. The study protocol adhered to the tenets of the Declaration of Helsinki and was reviewed and approved by the Institutional Review Board of the Seoul National University Hospital (IRB no: 1901-159-1006). This study was registered at ClinicalTrials.gov (Identifier NCT04130841) on 17/10/2019. Written informed consent for participation was obtained from all patients before enrollment. Subject recruitment was conducted between September 2019 and September 2020, and the last enrolled patient completed a 12-month visit in September 2021.

Patients scheduled for vitrectomy for the treatment of symptomatic idiopathic ERM confirmed by funduscopic examination and spectral-domain optical coherence tomography (OCT) were assessed for eligibility. Only patients older than 18 years with best-corrected visual acuity (BCVA) of ≤ 90 Early Treatment Diabetic Retinopathy Study (ETDRS) letters were included. Exclusion criteria were as follows: (1) ERM secondary to other retinal diseases, such as retinal vascular diseases, intraocular inflammation, retinal detachment, or trauma; (2) history of intraocular surgery except uncomplicated cataract surgery; (3) high myopia with axial length ≥ 26 mm and/or spherical equivalent ≥ -6 diopters; and (4) any concomitant ocular or retinal comorbidity affecting visual function such as glaucoma, central serous chorioretinopathy, and exudative age-related macular degeneration.

### Surgical procedures and randomization

All surgeries were performed by a single experienced surgeon (U.C.P.). The patients were treated with a standard three-port 23-gauge PPV under general or retrobulbar anesthesia. Combined phacoemulsification and intraocular lens implantation were performed in eyes with visually significant cataracts. After core vitrectomy, posterior vitreous detachment was induced, if required. All visible ERM was removed to the vascular arcades using end-gripping forceps, and the macular area was stained with 0.05% ICG dye diluted with 5% dextrose for 10 s to assess the ILM status. The pattern of residual ILM at the macular area was classified into four types: pattern A, the ILM was mostly removed within the vascular arcades; pattern B, the ILM was partially removed, but no residual ILM was intact within a 1-disc area centered on the foveal center; pattern C, the ILM was partially removed, and any residual ILM was intact within a 1-disc area centered on the foveal center; and pattern D, the ILM remained completely intact. Patients with pattern A were allocated to group 1 (involuntary peeling), while those with patterns B, C, and D were electronically randomized in the operating room in a 1:1 ratio to the active peeling (group 2) or non-peeling (group 3) of the residual ILM (http://www.randomization.com). In group 2, the residual ILM was peeled off to the vascular arcades. All patients received standard postoperative care with topical antibiotics and anti-inflammatory medications.

### Ophthalmic examinations

After ERM surgery, patient visits were scheduled at 1 day, 1 week, and 1, 3, 6, and 12 months. All patients underwent a comprehensive ophthalmologic examination at the preoperative and postoperative visits, and examiners were blinded to the group assignment. Ophthalmologic examinations included slit-lamp biomicroscopy, dilated fundus examination, BCVA, intraocular pressure, metamorphopsia using M-CHARTS (Inami Co., Tokyo, Japan)^17^, aniseikonia using the New Aniseikonia Test (NAT version 3; Handaya, Tokyo, Japan)^18^, spectral-domain OCT, and OCT angiography. The ETDRS chart was used for BCVA measurement. The horizontal and vertical scores were averaged for the analysis of metamorphopsia and aniseikonia measurements. Aniseikonia evaluation was performed only in patients without any pathological condition that could influence vision in the macula of the fellow eyes. Spectral-domain OCT examination was performed using Cirrus HD-OCT (Carl Zeiss Meditec, Dublin, CA, USA) with a 6 × 6 mm macular cube with a 200 × 200 scan and an HD 5-line raster scan. The mean retinal thicknesses of the nine macular sectors, as defined by the ETDRS, were recorded, and central macular thickness (CMT) was defined as the central 1-mm subfield thickness in the ETDRS grid map. The ERM severity was graded according to Govetto et al.’s ectopic inner foveal layer (EIFL) classification scheme^19^. Additionally, 12 months after surgery, a multifocal electroretinogram (mfERG) was performed (RETI-scan; Roland Consult, Stasche & Finger GmbH, Wiesbaden, Germany) according to the standard document of the International Society for Clinical Electrophysiology of Vision for clinical mfERG by an experienced investigator using contact lens electrodes^20^. Individual mfERG responses for the hexagons were grouped into six concentric rings centered on the fovea (ring 1 representing the < 1.7° field; ring 2, 1.7–5.6°; ring 3, 5.6–10.2°; ring 4, 10.2–15.6°; ring 5, 15.6–21.7°; and ring 6, 21.7–28.6°). The amplitudes and implicit times of the P1 and N1 responses were averaged for each ring.

### Primary and secondary outcome measures

The primary outcome measure was the ERM recurrence rate during the 12-month follow-up for each group. Recurrence of ERM was defined as the development of hyperreflective tissue on the inner surface of the retina which had disappeared after surgery, resulting in an increase in its area or thickness with focal thickening or wrinkling of the underlying retina compared to earlier postoperative OCT scans^21^. Simple reappearance of a thin hyperreflective membrane without change of macular contour was not considered ERM recurrence. The ERM recurrence was assessed by two blinded investigators (C.K.Y. and E.K.L.) who analyzed the OCT images independently. Any discrepancies were resolved by a senior investigator (U.C.P.). Secondary outcome measures included changes in BCVA, metamorphopsia score, aniseikonia score, CMT, and retinal thicknesses of the nine ETDRS macular sectors during a 12-month follow-up, and mfERG parameters at 12 months after surgery in each group.

### Sample size and statistical analysis

The sample size was calculated to highlight the difference in the ERM recurrence rate between active peeling (group 2) and non-peeling of the ILM (group 3) because patients with involuntary peeling (group 1) were not randomized. We assumed a difference of 21% in the recurrence rate based on previous reports^6,22^. To obtain 80% power with a type 1 error of 5%, 36 patients per group were required. Considering that spontaneous peeling of the ILM reportedly occurs in 30% of patients^23,24^, and that the remaining patients were to be randomized, the total number of patients required was 103. Allowing for a 20% drop-out rate, 129 patients were required to be enrolled for this study.

Continuous variables are reported as the mean ± standard deviation and categorical variables as frequency and percentage. Analysis of variance (ANOVA), Kruskal-Wallis test, and Mann–Whitney U test were used to compare continuous variables, and the Chi-square test was used to compare categorical variables among the groups. The Wilcoxon signed-rank test was used to compare continuous variables at different time points within each group. Patients who failed to follow up until 12 months after PPV were excluded from the analysis. Statistical significance was set at *P* < 0.05. Analyses were performed using SPSS software (version 23.0; IBM Corp., Armonk, NY, USA).

## Results

**Fig. 1:**
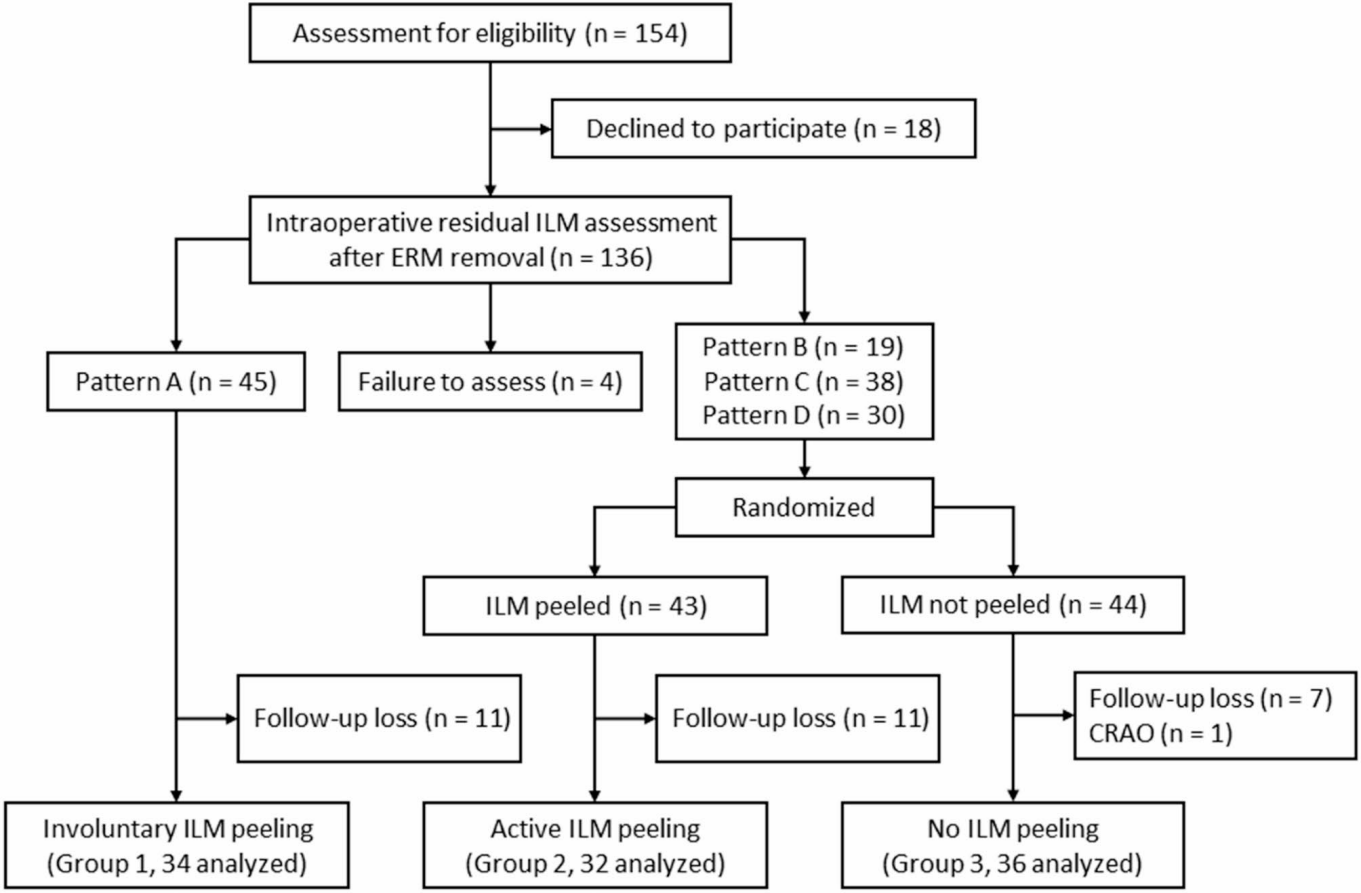
Flowchart illustrating the allocation process. Patients were classified into four patterns according to the status of the residual internal limiting membrane (ILM) after epiretinal membrane removal (see methods). Patients with complete ILM peeling (pattern A) were allocated to Group 1 (involuntary peeling), while those with partially removed or intact ILM (pattern B to D) were randomized in a 1:1 ratio to Group 2 and 3 (active peeling and non-peeling of residual ILM, respectively).* ILM* internal limiting membrane,* ERM* epiretinal membrane,* CRAO* central retinal artery occlusion.

During the study period, 154 patients who underwent PPV for idiopathic ERM removal met the inclusion criteria; however, 18 patients declined to participate. Residual ILM status at the macula just after ERM removal during surgery was classified as pattern A in 45 patients, pattern B in 19 patients, pattern C in 38 patients, and pattern D in 30 patients, while four patients were excluded due to assessment failure. Among patients with partially removed or intact ILM (patterns B to D), 43 and 44 patients were randomized to active peeling and non-peeling of the residual ILM during surgery, respectively. During follow-up, 30 patients were excluded: (1) 29 patients were lost to follow-up and (2) one patient developed central retinal artery occlusion during follow-up. Finally, 102 eyes (102 patients) were included in the analysis and classified as follows: 34 eyes (33.3%) in group 1 (involuntary peeling of ILM), 32 eyes (31.4%) in group 2 (active peeling of residual ILM), and 36 eyes (35.3%) in group 3 (non-peeling of residual ILM) (Fig. 1).

The demographic and baseline characteristics of the three groups are summarized in Table 1. There were no significant differences in age at the time of surgery, baseline BCVA, metamorphopsia, aniseikonia, EIFL grade, or CMT between the groups (*p* = 0.108, 0.859, 0.694, 0.141, 0.596, and 0.853, respectively). At the time of surgery, 8 (23.5%), 6 (18.8%), and 2 (5.6%) patients were pseudophakic in groups 1, 2, and 3 (*p* = 0.098), respectively. Combined cataract surgery at the time of PPV was performed in 25 (73.5%), 23 (71.9%), and 33 (91.7%) patients in groups 1, 2, and 3 (*p* = 0.077), respectively, and two patients in group 2 underwent cataract surgery during the 12-month follow-up period.

**Table 1:** Baseline demographics and clinical data of patients.

		Group 1 (*n* = 34)	Group 2 (*n* = 32)	Group 3 (*n* = 36)	*P* value
Age (year)		69.7 ± 8.1	66.1 ± 8.1	66.4 ± 7.0	0.108
Sex (M: F)		12 : 22	10 : 22	8 : 28	0.465
Diabetes mellitus (%)		7 (20.6)	7 (21.9)	6 (16.7)	0.851
Hypertension (%)		15 (44.1)	15 (46.9)	14 (38.9)	0.794
Dyslipidemia (%)		11 (32.4)	4 (12.5)	11 (30.6)	0.124
Pseudophakia (%)		8 (23.5)	6 (18.8)	2 (5.6)	0.098
Axial length (mm)		23.58 ± 1.28	24.12 ± 1.63	23.87 ± 0.93	0.244
BCVA (ETDRS)		66.2 ± 10.8	64.7 ± 14.5	65.5 ± 9.4	0.859
CMT (µm)		431.9 ± 79.1	435.5 ± 66.3	441.1 ± 59.9	0.853
M-chart score (°)		0.49 ± 0.49	0.40 ± 0.32	0.47 ± 0.48	0.694
Aniseikonia (%)^a^		4.04 ± 3.57	5.86 ± 4.58	6.09 ± 4.22	0.141
EIFL	Grade 1 (%)	0 (0)	0 (0)	0 (0)	0.596
	Grade 2 (%)	13 (38.2)	15 (46.9)	15 (41.7)	
	Grade 3 (%)	16 (47.1)	9 (28.1)	13 (36.1)	
	Grade 4 (%)	5 (14.7)	8 (25.0)	8 (22.2)	
Pattern	A (%)	34 (100)	0 (0)	0 (0)	N/A
	B (%)	0 (0)	8 (25.0)	9 (25.0)	
	C (%)	0 (0)	12 (37.5)	17 (47.2)	
	D (%)	0 (0)	12 (37.5)	10 (27.8)	

Data are presented as mean ± standard deviation.

*BCVA* best-corrected visual acuity,* CMT* central macular thickness,* EIFL* ectopic inner foveal layer.

^a^ Aniseikonia was analyzed for unilateral affected eyes; 7 patients (6 with epiretinal membrane (ERM) and 1 with branch retinal artery occlusion) in group 1, 9 patients (6 with ERM, 1 with neovascular age-related macular degeneration, 1 with central retinal vein occlusion, and 1 with anophthalmos) in group 2, and 7 patients (7 with ERM) in group 3 were excluded due to pathologic condition in the macula of the fellow eyes.

Recurrence of ERM was observed in 13 (12.7%) eyes during the 12-month follow-up period (Table 2). The number of eyes with ERM recurrence differed significantly among the groups: 0 of 34 (0%) in group 1, 0 of 32 (0%) in group 2, and 13 of 36 (36.1%) in group 3 (*p* < 0.001). Among the 13 eyes in group 3 that showed ERM recurrence, nine had residual ILM status of pattern C (partially removed and intact residual ILM within a 1-disc area centered on the foveal center) and four had pattern D (completely intact ILM). In contrast, no ERM recurrence was observed in group 3 eyes with pattern B (partially removed, but no intact residual ILM within a 1-disc area centered on the foveal center). During the 12-month study period, no patient required reoperation to remove the recurred ERM.

**Table 2:** Recurrences according to group and pattern.

		Recurrence (%)	No recurrence (%)	Total
Group 1 (involuntary peeling)	Pattern A	0 (0)	34 (100)	34 (100)
Group 2 (active peeling)	Pattern B	0 (0)	8 (100)	8 (100)
	Pattern C	0 (0)	12 (100)	12 (100)
	Pattern D	0 (0)	12 (100)	12 (100)
	Subtotal	0 (0)	32 (100)	32 (100)
Group 3 (non-peeling)	Pattern B	0 (0)	9 (100)	9 (100)
	Pattern C	9 (52.9)	8 (47.1)	17 (100)
	Pattern D	4 (40.0)	6 (60.0)	10 (100)
	Subtotal	13 (36.1)	23 (63.9)	36 (100)
Total		13 (12.7)	89 (87.3)	102 (100)

Pattern A, no residual internal limiting membrane (ILM) after epiretinal membrane (ERM) removal; pattern B, partially removed but no intact residual ILM within a 1-disc area centered on the foveal center; pattern C, partially removed and intact residual ILM within a 1-disc area centered on the foveal center; pattern D, completely intact ILM after ERM removal.

**Fig. 2:**
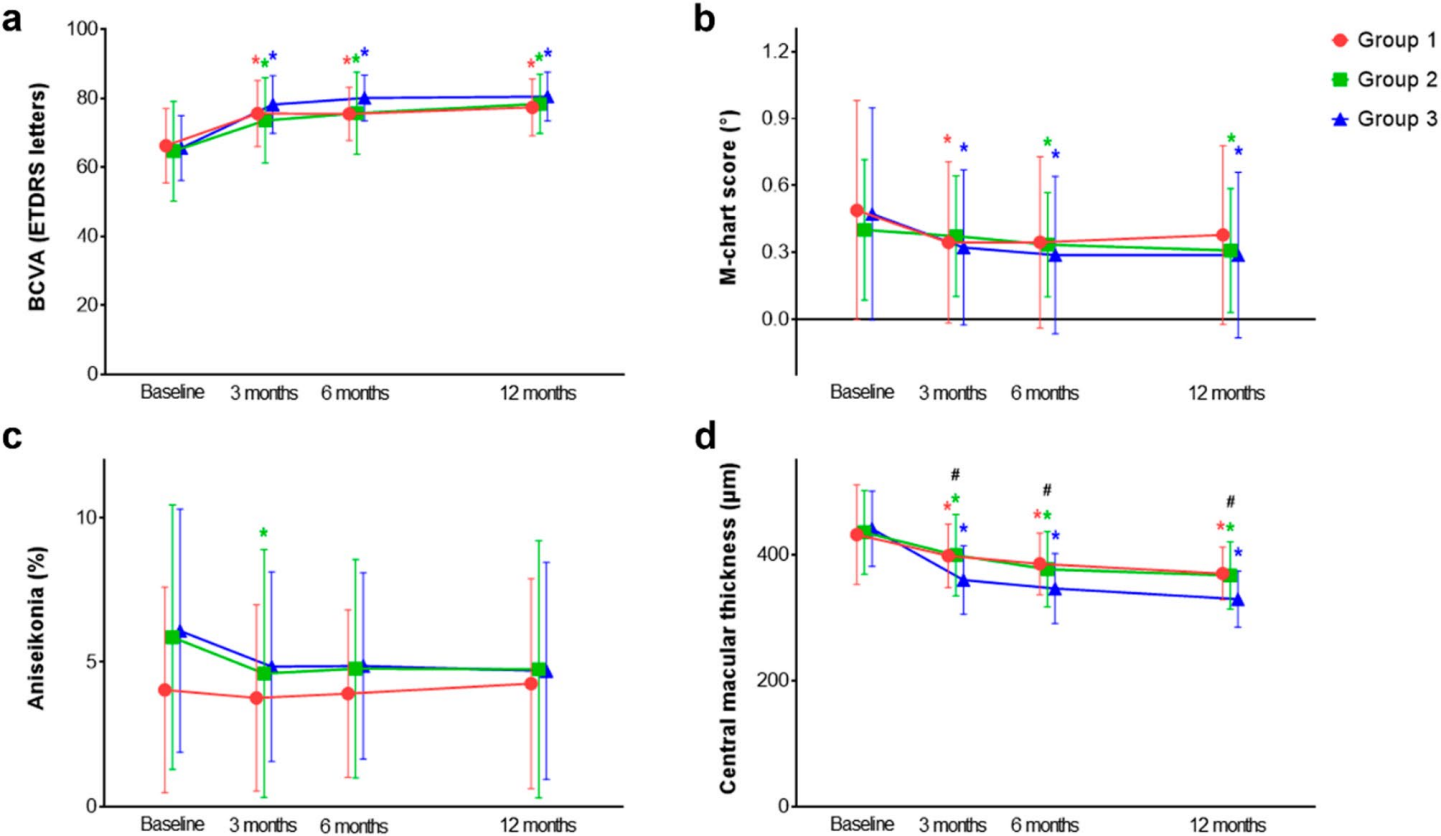
Changes in best-corrected visual acuity (**a**), metamorphopsia (**b**), aniseikonia (**c**), and central macular thickness (**d**) from baseline to 3, 6, and 12 months after surgery. Black hashes indicate significant differences among the three groups, and red, green, and blue asterisks indicate significant differences from the baseline at each time point for each group (*p* < 0.05). Error bar indicates standard deviation.

The changes in BCVA, metamorphopsia, aniseikonia, and CMT are shown in Fig. 2. At 3, 6, and 12 months after surgery, the mean BCVA improved significantly in all groups compared to that before surgery, and there was no difference in BCVA among the groups. The mean metamorphopsia scores of group 1 at 3 months, group 2 at 6 and 12 months, and group 3 at 3, 6, and 12 months after surgery showed a significant decrease from baseline; however, there was no significant difference in the metamorphopsia score among the groups. Aniseikonia showed no significant postoperative changes in any group except group 2 at 3 months after surgery, and there was no difference in the aniseikonia score among the groups. At 3, 6, and 12 months after surgery, all groups showed a significant decrease in CMT, and group 3 showed a significantly lower CMT than the other groups at all time points (*p* = 0.006, 0.012, and 0.001 at 3, 6, and 12 months, respectively).

**Fig. 3:**
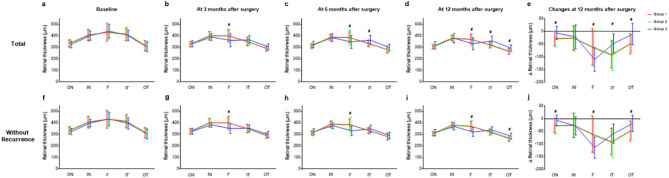
Retinal thicknesses of ETDRS macular sectors horizontally across the fovea. (**a-e**) Pre- and postoperative thickness profile of all patients. Changes in retinal thickness during the 12 months are shown in **e**. (**f-j**) Pre- and postoperative thickness profile of eyes without epiretinal membrane recurrence. Changes in retinal thickness during the 12 months are shown in **j**. Black hashes indicate significant differences among the three groups (*p* < 0.05). Error bar indicates standard deviation.*ON* outer nasal sector,* IN* inner nasal sector,* F* foveal sector,* IT* inner temporal sector,* OT* outer temporal sector.

When the retinal thicknesses of the ETDRS macular sectors were analyzed horizontally across the fovea, namely the outer nasal, inner nasal, foveal, inner temporal, and outer temporal sectors, the preoperative retinal thickness profile was comparable among the groups, showing the greatest thickness in the foveal sector and a gradual decrease toward the outer sectors (Fig. 3). Postoperatively, the retinal thickness at the foveal sector, namely the CMT, was significantly lower in group 3 than in the other groups at 3, 6, and 12 months (*p* = 0.006, 0.012, and 0.001, respectively). Group 3 showed a tendency towards postoperative restoration of foveal depression on average, showing that the foveal sector was thinner than the inner nasal and inner temporal sectors. In contrast, the other groups showed different patterns, with the greatest thickness in the inner nasal or foveal sector. There was no significant difference in retinal thickness in the inner and outer nasal sectors among the groups; however, the inner and outer temporal sectors in groups 1 and 2 were significantly thinner than those in group 3 at 12 months after surgery (*p* < 0.001 for both). The decrease in postoperative retinal thickness at 12 months was greatest in the foveal sector in group 3, whereas groups 1 and 2 showed the greatest decrease in the inner temporal sector. The decrease in the foveal sector in group 3 was significantly greater than that in groups 1 and 2, but the opposite was observed in the outer sectors and inner temporal sector. When the thickness profile at the horizontal ETDRS sectors was analyzed only for eyes without ERM recurrence, the foveal sector retinal thickness in group 3 was also significantly lower than that in the other groups at 3, 6, and 12 months after surgery (*p* = 0.002, 0.001, and < 0.001, respectively), and the outer temporal sector retinal thickness in groups 1 and 2 was significantly lower than that in group 3 at 12 months after surgery (*p* = 0.008).

The thickness profiles of the vertical ETDRS sectors across the fovea, namely the outer superior, inner superior, foveal, inner inferior, and outer inferior sectors, were also evaluated. The retinal thicknesses at the outer superior, inner superior, and inner inferior sectors in groups 1 and 2 were significantly lower than those in group 3 at 12 months after surgery (*p* < 0.001, = 0.012, and 0.004, respectively). When the thickness profile at the vertical ETDRS sectors was analyzed only for eyes without ERM recurrence, the retinal thickness at the outer superior sector in groups 1 and 2 was significantly lower than that in group 3 at 12 months after surgery (*p* = 0.003) (Supplementary Fig. S1 online). The decrease in postoperative retinal thickness at 12 months in groups 1 and 2 was significantly greater than that in group 3 in the outer sectors.

**Fig. 4:**
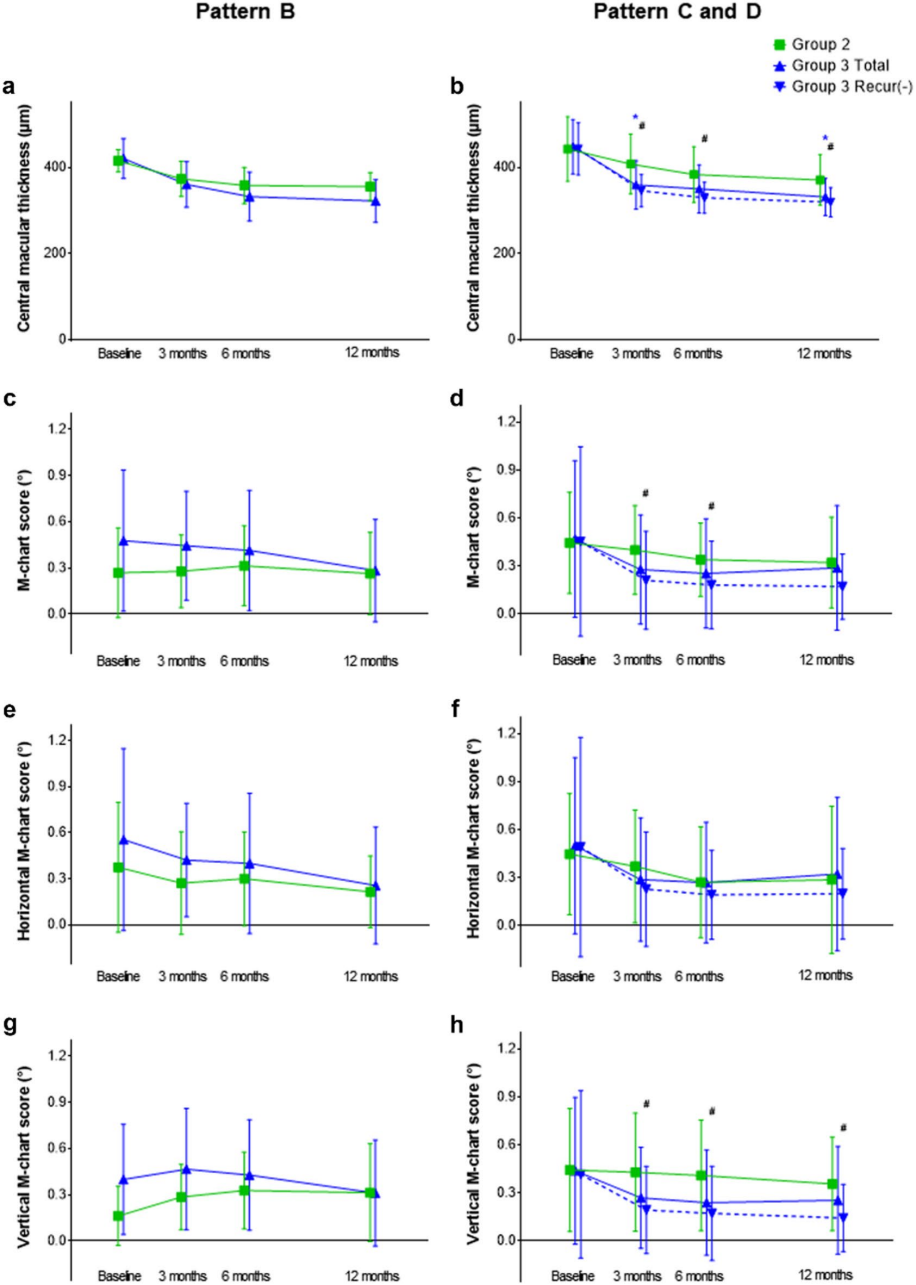
Postoperative changes in central macular thickness (**a**,** b**) and average, horizontal, and vertical metamorphopsia scores (**c**-**h**) according to the active peeling of residual ILM (group 2, active peeling; group 3, non-peeling) in eyes with residual ILM not involving the fovea (pattern B) and involving the fovea (pattern C and D). Blue asterisks indicate significant differences between Group 2 and Group 3, and black hashes indicate significant differences between Group 2 and Group 3 patients without ERM recurrence (*p* < 0.05). Error bar indicates standard deviation.

We performed an additional subgroup analysis in eyes with partially removed or completely intact ILM to investigate the influence of active peeling of residual ILM (Fig. 4). In eyes with residual ILM not involving the fovea (pattern B), there were no significant differences between groups 2 and 3 during follow-up for any of the parameters, including BCVA, metamorphopsia, aniseikonia, and CMT. However, in eyes with residual ILM involving the fovea (patterns C or D), the CMT of group 3 at 3 and 12 months after surgery was significantly lower than that of group 2 (*p* = 0.008 and 0.012, respectively). The difference was more prominent when cases with ERM recurrence, which were observed only in group 3, were excluded (*p* = 0.004, 0.014, and 0.006 at 3, 6, and 12 months, respectively). The metamorphopsia score in eyes with residual ILM not involving the fovea did not differ according to ILM peeling; however, in eyes with residual ILM involving the fovea, the metamorphopsia score was lower in group 3 at 3 and 6 months when recurrent cases were excluded (*p* = 0.030 and 0.041, respectively). When horizontal and vertical metamorphopsia scores were separately analyzed in eyes with residual ILM involving the fovea, only the vertical score for group 3 was significantly lower than that of group 2 when patients with ERM recurrence were excluded (*p* = 0.043, 0.024, and 0.034 at 3, 6, and 12 months, respectively), whereas the horizontal scores were comparable.

There were no significant differences among the groups in mfERG parameters, such as P1 amplitude, P1 implicit time, N1 amplitude, and N1 implicit time at 12 months after surgery for every ring (Supplementary Table S1).

## Discussion

In this randomized controlled clinical trial, we investigated how the ILM status and intentional active peeling of the residual ILM following ERM removal affected the surgical outcome of idiopathic ERM. Our findings demonstrate a significant decrease in ERM recurrence during 12 months after surgery in eyes with ILM peeling, with no recurrence in the groups wherein the ILM was involuntarily (group 1) or actively peeled (group 2). In contrast, ERM recurrence was observed only when the residual ILM was not peeled off (group 3), particularly when the residual ILM involved the fovea. The overall visual outcomes were comparable across the three groups; however, the decrease in CMT after surgery was significantly greater in the non-peeling group than in other groups. At 12 months after surgery, the non-peeling group showed restoration of the thickness profile similar to the normal macular contour than the involuntary or active peeling groups. In addition, a subgroup analysis of eyes with residual ILM involving the fovea showed that non-peeling of the ILM was associated with lower metamorphopsia, if ERM did not recur postoperatively, suggesting the potential benefits of non-peeling ILM.

Previous studies comparing ERM surgery with or without ILM peeling presented conflicting findings regarding the anatomical or functional influence of ILM peeling^5,11,25–31^, catalyzing a number of meta-analyses on this topic. Most meta-analyses reported the efficacy of double peeling of the ERM and ILM in significantly reducing ERM recurrence rates compared to ERM removal alone while demonstrating comparable visual outcomes^7,32–36^. Additionally, a recent meta-analysis including randomized controlled trials confirmed this finding^10^. Some meta-analyses observed a greater decrease in CMT after ILM peeling^10,36–38^, while others observed comparable thickness changes.

During ERM removal, the ILM is often peeled off simultaneously with the ERM, en bloc or partially. However, most previous studies comparing the results of ERM removal according to the peeling of the ILM have not mentioned the ILM status after ERM removal. Thus, main strength of the present study is that ILM integrity after ERM removal was considered in assessing the influence of active peeling of the ILM, which was compared in terms of ERM recurrence, anatomical changes, and visual function including metamorphopsia and aniseikonia. In this study, immediately after ERM removal during surgery, the ILM was mostly peeled up to the vascular arcade in 45 of 136 (33.1%) eyes, partially peeled in 57 of 136 (41.9%) eyes, and completely intact in 30 of 136 (22.1%) eyes. These proportions are comparable to results from previous studies in which complete peeling (en block removal) of ILM simultaneously during the ERM removal was reported in 31.0–65.4% of cases and undamaged intact ILM was observed in 3.8–45.9%^15,16,23,24,28,39,40^. Occurrence of simultaneous ILM peeling during ERM removal appears to be influenced by the broadness of adhesion between the ERM and inner retina visible on OCT image^39^ and adhesive cellular proliferation between the ERM and ILM at microscopic level^24^. However, functional and anatomical outcomes were similar between the involuntary and active peeling groups in the present study, which suggests that the clinical impact of active peeling of ILM may not be different from its spontaneous peeling with ERM.

In this study, ERM recurrence within 12 months after surgery was observed only in group 3, the non-peeling group, confirming the results from the majority of previous studies^5,6,8,22,40,41^. Notably, recurrence was observed only when completely intact ILM (pattern D) or partially removed ILM remaining at the fovea (pattern C) was not peeled off, suggesting that residual ILM involving the fovea is important for recurrence. When the ILM was present despite ERM removal, the presence of ERM fragments along with glial cells, hyalocytes, and myofibroblasts was observed on the ILM^6,8,16^. In a histopathologic study, an average of 20% of the total cell count comprising the ERM, ranging from 2 to 51%, was found to be left behind on the ILM when the ERM was removed only^24^. In addition, the ILM is assumed to serve as a scaffold for cell proliferation^6^. This means that complete peeling of the ILM may prevent cellular proliferation, lowering ERM recurrence.

Patients with non-peeling of the residual ILM (group 3) showed a significantly greater decrease in CMT compared to other groups with involuntary or active peeling of the residual ILM (group 1 or 2, respectively). In particular, the retinal thickness pattern at each ETDRS sector showed the greatest decrease in the foveal sector at 12 months after surgery, showing recovery to the normal contour of the macula, with the foveal sector being thinner than the inner nasal and inner temporal sectors. In contrast, in the other two groups of ILM peeling, which had similar thickness patterns, regardless of involuntary or active peeling, the greatest thickness decrease was observed in the inner temporal sector, while the nasal sectors showed less decrease than other horizontal sectors. This resulted in thickness patterns differing from those in group 3, with the foveal and inner nasal sectors being thicker than the other sectors at 12 months after surgery. This thickness pattern observed in the two ILM peeling groups corresponds to nasal crowding and nasal shift of the fovea, which are found after ERM removal with ILM peeling^42–44^. This finding suggests that intact ILM may be associated with the recovery of normal macular contour, although peeling of the residual ILM decreases ERM recurrence. The mechanism for different postoperative thickness patterns according to ILM peeling during ERM surgery is unclear; however, it is assumed that maintenance of the Müller cell structure by non-peeling of ILM may prevent nasal crowding secondary to centrifugal expansion of the retinal tissue and contraction of the retinal nerve fiber layer^11,12,44,45^.

Regarding visual function in terms of BCVA, metamorphopsia, and aniseikonia, group 3 showed no significant differences compared to other groups. In the subgroup analysis, a comparison between active peeling and non-peeling of the residual ILM was performed separately in eyes with residual ILM not involving the fovea (pattern B) and those with residual ILM involving the fovea (pattern C or D). Along with a lower CMT, significantly lower metamorphopsia scores were observed in the non-peeling group only in eyes with residual ILM involving the fovea, especially when eyes with ERM recurrence were excluded. This seems to be associated with the recovery of the relatively normal contour of the macula in eyes in which the residual ILM was preserved. Notably, only the vertical metamorphopsia score was significantly different according to active peeling of the ILM in eyes with residual ILM involving the fovea but without ERM recurrence, whereas horizontal metamorphopsia was comparable. Nasal crowding in eyes with ILM peeling probably hindered the improvement of vertical metamorphopsia in comparison to those without ILM peeling, while postoperative changes in horizontal metamorphopsia scores were similar between the groups. This finding corresponds to the results of the square grid analysis of macular deformation after macular hole surgery with ILM peeling, which showed that vertical and horizontal metamorphopsia correlated with the deformation of the vertical and horizontal lines, respectively^45^.

In the present study, functional assessment using mfERG did not show significant differences among the groups at 12 months after surgery. This suggests that the different management of residual ILM did not significantly affect the focal retinal function assessed by mfERG and supports the interpretation that the observed changes in retinal thickness and metamorphopsia are not due to focal functional damage of the retina. This is in line with a previous study that found no significant difference in P1 amplitude and peak time between patients who underwent ERM removal alone and those who underwent additional ILM peeling^46^.

Nevertheless, this study had some limitations. First, microperimetry was not performed, and retinal sensitivity and the presence of microscotoma could not be evaluated. Even with good visual acuity, decreased retinal sensitivity and deep microscotomas may induce visual discomfort; hence, microperimetry would be useful in assessing microscotoma caused by ILM peeling^23,40,47^. In a recent prospective randomized trial, microperimetric outcomes were compared between active and non-peeling of ILM when its spontaneous peeling did not occur after ERM removal, and only non-peeling group showed significant improvement in retinal sensitivity postoperatively while active peeling group showed greater number of scotoma at one month after surgery^40^. Second, ICG toxicity could have affected retinal structure and function, more likely when the ILM was peeled off simultaneously during ERM removal and the retinal tissue was exposed. This may serve as a bias towards worse anatomical and functional outcomes in the involuntary peeling group, and the ICG dye was used for only 10 s to minimize this possibility. Third, not all phakic eyes at the time of surgery underwent combined cataract surgery and this can be a possible source of bias for visual acuity, although only 1 in 34, 3 in 32, and 1 in 36 patients in groups 1, 2, and 3, respectively, remained phakic after surgery. Fourth, mfERG was later determined to be performed at 12 months after surgery, thus, the postoperative change could not be assessed due to the absence of preoperative data. Fifth, the rate of follow-up loss was relatively high due to patients being unable to attend scheduled visits during the COVID-19 pandemic. This limitation may have introduced a selection bias, potentially affecting the generalizability of the findings. Lastly, the subgroup analyses excluding eyes with ERM recurrence resulted in a decreased sample size in the group 3, but appropriate non-parametric statistical methods were applied to ensure valid interpretation.

In conclusion, this study provides valuable insights into the role of ILM peeling based on its integrity after ERM removal in patients with idiopathic ERM. Our findings confirmed the previous notion that ILM peeling is associated with a lower rate of ERM recurrence. However, structural recovery of the macula was more favorable when the residual ILM was left unpeeled, which seems to be associated with less severe metamorphopsia during postoperative follow-up when ERM did not recur. Consequently, peeling of the residual ILM during ERM surgery should be carefully performed considering these aspects. If ERM recurrence can be prevented with a certain method, non-peeling of the residual ILM during ERM surgery could be considered a better surgical option, enabling greater anatomical and functional improvement.

## Electronic supplementary material

Below is the link to the electronic supplementary material.

Supplementary Material 1

Supplementary Material 2

## Data Availability

The datasets generated during and/or analysed during the current study are available from the corresponding author on reasonable request.
