# Supplementary material for: Influence of internal limiting membrane peeling during idiopathic epiretinal membrane removal: a randomized controlled trial
Source: Sci Rep. 2025 May 20;15:17499. doi: 10.1038/s41598-025-01987-z (PMC12092785; doi:10.1038/s41598-025-01987-z)
Supplement: Supplementary file 1 — Supplementary Material 1 [file 41598_2025_1987_MOESM1_ESM.pdf]

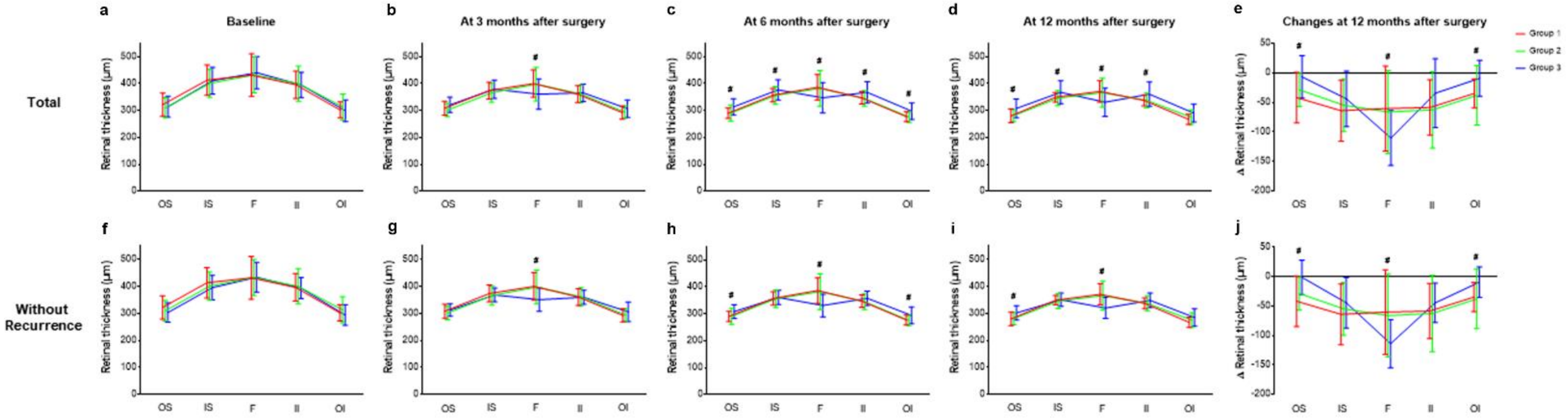

Supplementary Fig. S1. Retinal thicknesses of ETDRS macular sectors vertically across the fovea. (**a-e**) Pre- and postoperative thickness profile of all patients. Changes in retinal thickness during the 12 months are shown in **e**. (**f-j**) Pre- and postoperative thickness profile of eyes without epiretinal membrane recurrence. Changes in retinal thickness during the 12 months are shown in **j**. Black hashes indicate significant differences among the three groups ($p < 0.05$). Error bar indicates standard deviation.

OS, outer superior sector; IS, inner superior sector; F, foveal sector; II, inner inferior sector; OI, outer inferior sector.
