# Supplementary material for: Influence of internal limiting membrane peeling during idiopathic epiretinal membrane removal: a randomized controlled trial
Source: Sci Rep. 2025 May 20;15:17499. doi: 10.1038/s41598-025-01987-z (PMC12092785; doi:10.1038/s41598-025-01987-z)
Supplement: Supplementary file 2 — Supplementary Material 2 [file 41598_2025_1987_MOESM2_ESM.pdf]

Supplementary Table S1. Multifocal electroretinogram data at 12 months after surgery

		Group 1	Group 2	Group 3	*P* value
P1 amplitude	ring 1	86.97 ± 44.49	80.04 ± 39.96	84.52 ± 50.85	0.866
	ring 2	51.91 ± 21.34	53.82 ± 16.01	56.89 ± 18.44	0.650
	ring 3	35.31 ± 10.29	41.15 ± 10.06	41.04 ± 11.93	0.108
	ring 4	24.31 ± 7.93	28.28 ± 7.30	26.91 ± 7.38	0.187
	ring 5	19.03 ± 5.76	22.25 ± 6.20	19.29 ± 7.58	0.175
	ring 6	14.93 ± 5.12	16.89 ± 5.24	15.95 ± 6.20	0.475
P1 time	ring 1	43.35 ± 5.96	41.60 ± 6.06	44.05 ± 4.71	0.303
	ring 2	40.57 ± 3.41	41.06 ± 2.88	41.11 ± 2.75	0.790
	ring 3	40.13 ± 2.82	41.25 ± 1.83	41.39 ± 2.61	0.157
	ring 4	41.53 ± 3.14	41.18 ± 1.81	41.80 ± 2.28	0.686
	ring 5	41.96 ± 2.10	42.42 ± 1.97	42.07 ± 2.14	0.722
	ring 6	42.65 ± 2.14	43.31 ± 1.82	42.93 ± 2.20	0.541
N1 amplitude	ring 1	30.52 ± 21.67	43.60 ± 42.84	37.66 ± 29.41	0.383
	ring 2	18.87 ± 9.53	18.66 ± 9.33	20.42 ± 12.75	0.823
	ring 3	13.76 ± 5.23	15.52 ± 7.07	12.75 ± 4.73	0.247
	ring 4	8.54 ± 3.79	10.11 ± 3.30	8.36 ± 3.80	0.196
	ring 5	6.70 ± 3.04	7.45 ± 2.28	6.44 ± 2.18	0.364
	ring 6	5.88 ± 2.46	6.13 ± 1.82	5.33 ± 2.12	0.421
N1 time	ring 1	22.91 ± 5.53	23.03 ± 4.73	22.53 ± 5.22	0.940
	ring 2	23.65 ± 2.55	24.73 ± 2.40	23.92 ± 2.28	0.277
	ring 3	23.45 ± 2.28	23.61 ± 2.34	23.07 ± 2.30	0.705
	ring 4	23.72 ± 2.09	24.42 ± 2.22	22.64 ± 3.82	0.096
	ring 5	23.72 ± 2.37	24.15 ± 1.82	24.81 ± 2.00	0.198
	ring 6	25.51 ± 2.05	25.70 ± 1.44	24.42 ± 2.63	0.083

Data are presented as mean ± standard deviation.
